# Ultrasound variants of autoimmune thyroiditis in children and adolescents and their clinical implication in relation to papillary thyroid carcinoma development

**DOI:** 10.1007/s40618-017-0758-z

**Published:** 2017-09-02

**Authors:** D. Januś, M. Wójcik, G. Drabik, Ł. Wyrobek, J. B. Starzyk

**Affiliations:** 1 Department of Pediatric and Adolescent Endocrinology, Chair of Pediatrics, Institute of Pediatrics, Jagiellonian University Medical College, Wielicka St. 265, 30-663 Krakow, Poland; 2 Department of Pediatric and Adolescent Endocrinology, University Children Hospital, Krakow, Poland; 3 Department of Clinical Immunology and Transplantation, Institute of Paediatrics, Jagiellonian University Medical College, Krakow, Poland; 4 Department of Radiology, University Children Hospital, Krakow, Poland

**Keywords:** Autoimmune thyroiditis, Normoechogenic thyroid background, Normoechogenic thyroid parenchyma, Papillary thyroid carcinoma, aTPO, aTG

## Abstract

**Background:**

The prevalence of autoimmune thyroiditis (AIT) and papillary thyroid carcinoma (PTC) is rising in children and adolescents, and the coincidence of AIT and PTC is as high as 6.3–43%.

**Objective:**

To investigate the ultrasound manifestation of AIT in relation to PTC development in paediatric patients.

**Patients:**

179 paediatric patients (133 females), mean (SD) age: 13.9 (3.03) years diagnosed with AIT and referred for ultrasound evaluation. Eight patients were diagnosed with PTC (6 females).

**Methods:**

Retrospective analysis of thyroid ultrasound scans of patients diagnosed with AIT. Thyroid and autoimmune status was assessed based on TSH, fT4, fT3 and increased aTPO and/or aTG and/or TRAB levels. In patients with PTC, total thyroidectomy was performed.

**Results:**

Analysis of thyroid US scans revealed that the following five ultrasound variants of AIT were observed in 179 patients: the most common in 35.2%—diffuse thyroiditis with hypoechogenic background and normoechogenic parenchyma, in 30.2%—diffuse thyroiditis with irregular background, in 18.9% nodular variant with normoechogenic background, in 11.7%—micronodulations and in 3.9%—diffuse hypoechogenic background. Eight cases of PTC were diagnosed in nodular variant of AIT with normoechogenic irregular background.

**Conclusion:**

Patients with AIT and nodular variant with normoechogenic irregular background of the thyroid gland on US scans are in the risk group of developing PTC and should be followed up with regular neck US assessment.

## Introduction

The prevalence of chronic autoimmune thyroiditis (AIT) has been assessed as 0.3–2% in children and 4–9.6% in adolescents and is rising [1, 2]. The childhood prevalence of AIT peaks in early to mid-puberty, with a female preponderance of 2:1 [3]. Presentation is rare under the age of 3 years, but few cases have been described in infancy [4]. AIT is considered to be a premalignant lesion, with an increased prevalence in papillary thyroid cancer [5–7].

Papillary thyroid carcinoma (PTC) accounts for 90% or more of all childhood differentiated thyroid carcinoma (DTC) cases [8]. The prevalence of PTC is rising in the last years partly due to more frequent ultrasound (US) assessments and US controlled fine needle aspiration biopsy (FNAB) of small nodules of the thyroid gland. The incidence of thyroid nodules in children and adolescents is 1–18% [9]. The risk of thyroid cancer in children operated due to nodular goiter is significantly higher than in adults (~25% of children operated) [8].

In the last years, there is an increase of coincidence of AIT and PTC in children and adolescents. A coincidence of AIT and PTC is ranging between 6.3 and 43%, depending on the patients selection [10–12]. Oh et al. [13] presented that in patients with PTC, the prevalence of chronic lymphocytic thyroiditis increased fourfold in men and twofold in women between 1999 and 2008. In pediatric patients with PTC, Niedziela et al. [14] found that the prevalence of chronic lymphocytic thyroiditis increased tenfold between 1996–2000 and 2001–2015 years.

According to the current paediatric guidelines neck US in children with autoimmune thyroid disease should be performed at least every 12 months [8, 15]. Knowing how different ultrasound pictures of the thyroid gland in AIT patients are observed, the question then arises if PTC can be associated with certain type of autoimmune thyroiditis variant and whether US evaluation of patients with AIT can help in selecting a risk group.

The aim of the present study is a trial to classify US thyroid scans in AIT patients and to assess characteristic features of thyroid presentation in patients with PTC and AIT.

## Subjects and methods

### Subjects

The study included 179 paediatric patients (133 females, 46 males; female-to-male ratio: 2.9:1) diagnosed with AIT and referred for ultrasound evaluation in the Out-Patient Endocrine Department between years 2015 and 2016. The mean age of the patients was 13.9 years (range 5–18 years). Eight patients were diagnosed with papillary thyroid carcinoma (6 females and 2 males, mean age, range 15.3 [11–18] years).

### Methods

Retrospective analysis of medical records and thyroid US scans of 179 patients diagnosed with AIT between 2015 and 2016 in the major tertiary paediatric endocrinology center was performed.

The analyzed data were as follows: age at diagnosis, gender, thyroid status (euthyroid, hypothyroid, hyperthyroid), autoantibodies levels (thyroperoxidase, aTPO; thyroglobuline, aTG; and TSH receptor, TRabs, antibodies) and US features of the AIT. Autoimmune thyroid disease (AITD) was diagnosed based on clinical (presence of goiter, firm consistency of the thyroid gland) and hormonal (TSH, fT4, fT3 levels) parameters as well as typical features of chronic autoimmune thyroiditis on thyroid ultrasound scans and increased aTPO, and/or aTG and/or TRab levels. Compensated (or subclinical) hypothyroidism was diagnosed if TSH was above the upper normal range (TSH *n*: 0.4–4.0 uIU/ml) and fT4 close to low normal range (fT4 *n*: 10–25 pmol/l). Overt hypothyroidism was diagnosed if TSH was above the upper normal range and fT4 was below the low normal range. Overt hyperthyroidism was diagnosed if TSH was suppressed and fT4 and fT3 above the upper normal range (fT3 *n*: 3.8–6.8 pmol/l). AIT was diagnosed in euthyroid patients with an increased aTPO and/or aTG and/or TRab antibody levels and typical features of chronic autoimmune thyroiditis on thyroid ultrasound scans.

### Ultrasound thyroid evaluation

US of the thyroid gland was performed in the Out-Patient Endocrine Department of the University Children Hospital by one board US certified endocrinologist with 17 years of experience in pediatric thyroid imaging and intervention (fine needle aspiration biopsy). Thyroid US was performed using a high-resolution Voluson 730, GE Medical System with an 8- to 12-MHz linear transducer. The US examination was performed in the longitudinal and axial planes. Scans indicating background parenchymal echogenicity were reviewed retrospectively. Normal thyroid parenchyma (normoechogenic background) was defined as demonstrating homogenous echogenicity and relative hyperechogenicity compared with the adjacent sternohyoid, sternothyroid, omohyoid and sternocleidomastoid muscles. The abnormal parenchymal features of the thyroid gland, including irregular echotexture, micronodularity, and diffuse or focal hypoechogenic lesions and nodules on gray-scale US, were evaluated. FNAB was performed in 34 patients with a nodule found on US.

### Statistical analysis

To compare the two groups, the two-sided Mann–Whitney *U* test and ANOVA tests were used. For comparison of multiple groups, ANOVA Kruskal–Wallis test was used. The level of significance was set at *p* < 0.05. Calculations were performed using the STATISTICA 10.0 PL software (Poland).

## Results

**Fig. 1:**
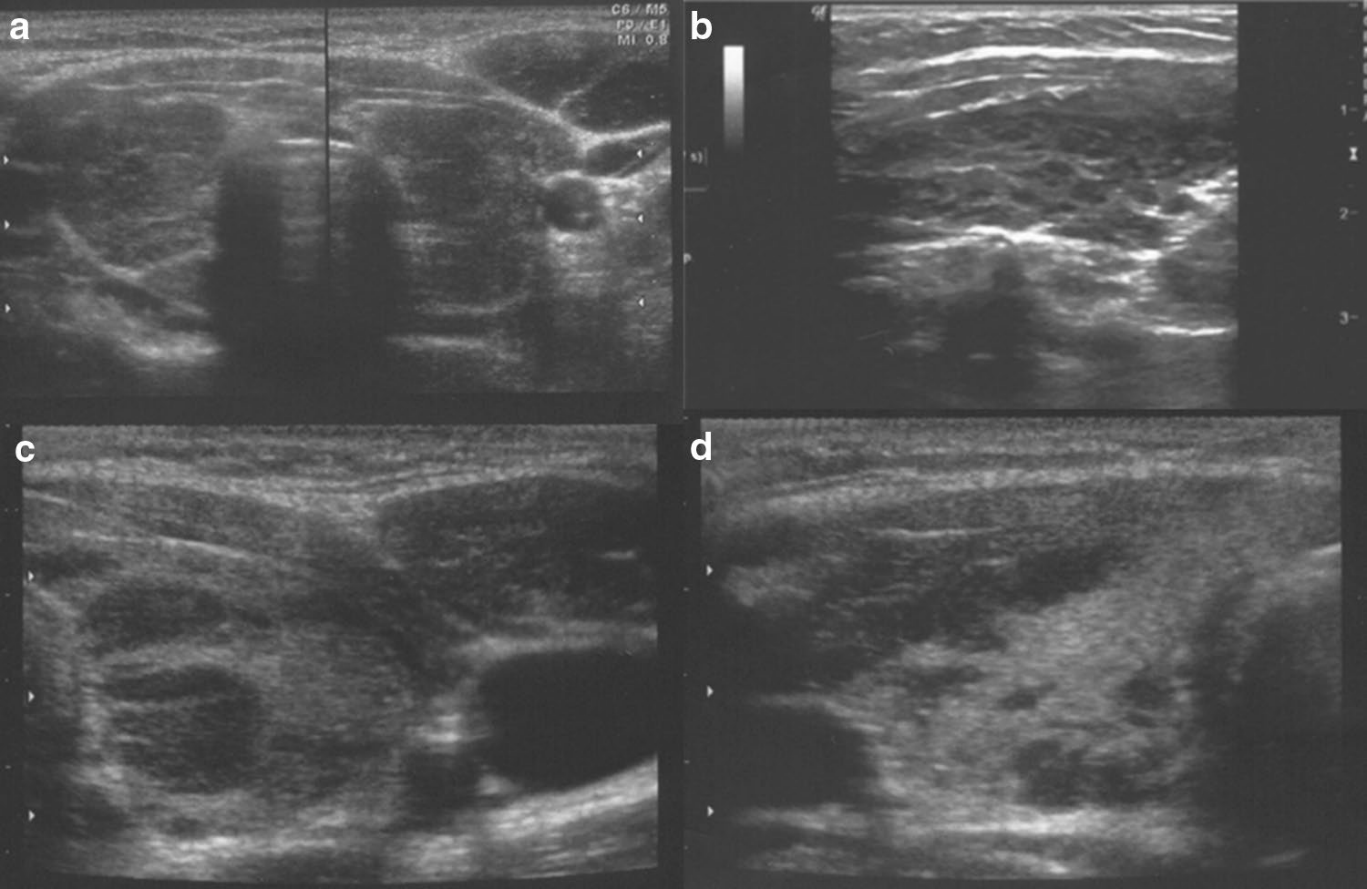
**a**, **b** Diffuse thyroiditis with hypoechogenic background and normoechogenic parenchyma, **c**, **d** diffuse thyroiditis with irregular background

**Fig. 2:**
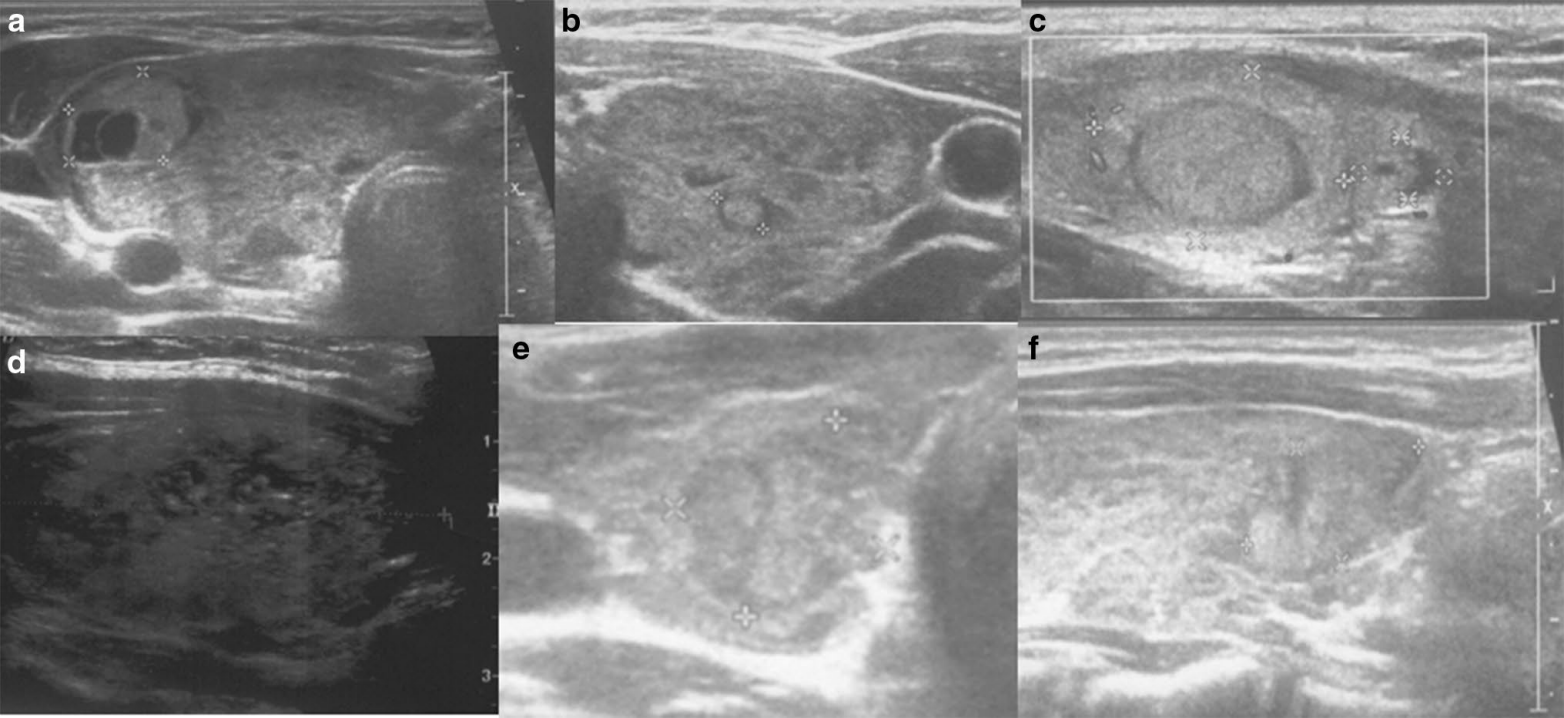
Nodular AIT variants with normoechogenic irregular background with PTC development. **a**, **b** Axial planes of thyroid gland of 17-year-old girl; **c** longitudinal plane of thyroid gland of 11-year-old boy; **d** an axial plane of thyroid gland of 18-year-old girl; **e**, **f** axial and longitudinal planes of thyroid gland of 17-year-old girl

**Fig. 3:**
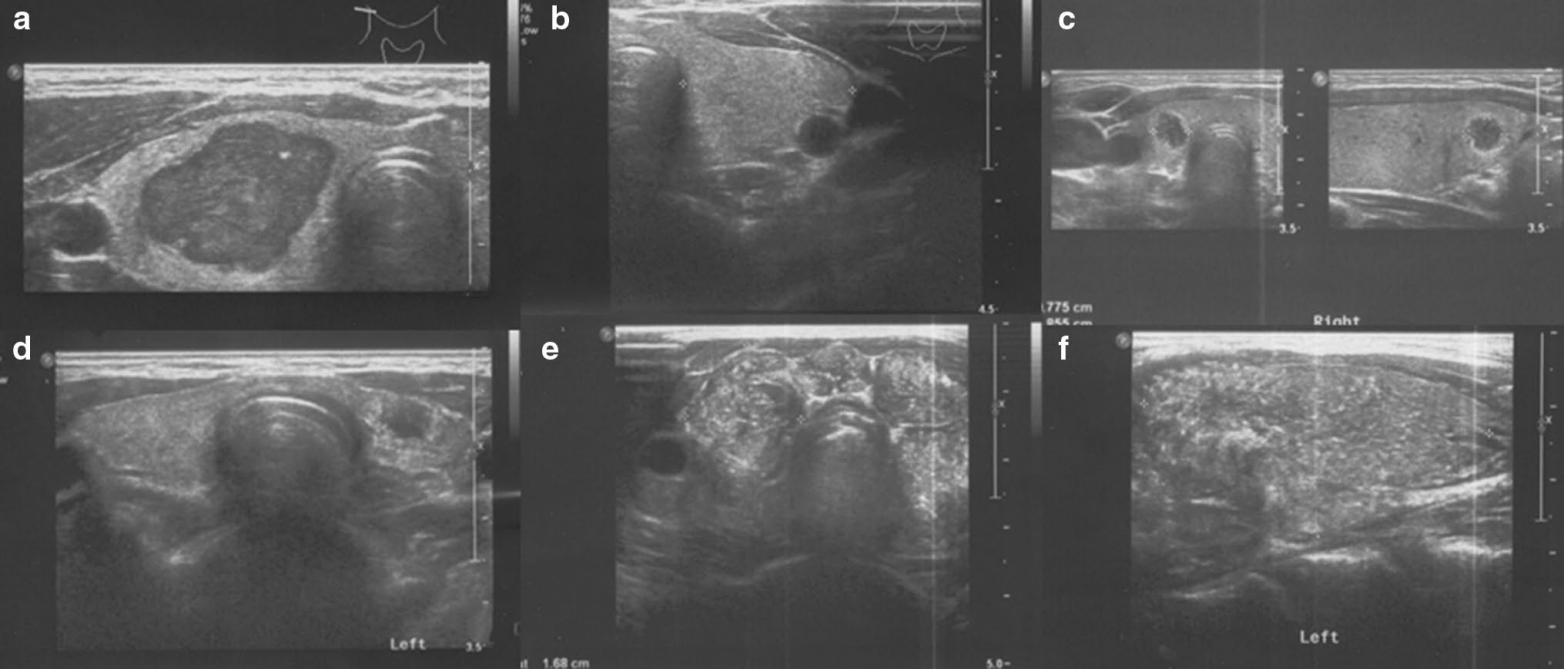
Nodular AIT variants with normoechogenic irregular background with PTC development. **a**, **b** Axial planes of thyroid gland of 18-year girl; **c** axial and longitudinal plane of thyroid gland of 14.5-year-old girl; **d** an axial plane of thyroid gland of 14-year-old girl; **e**, **f** axial and longitudinal planes of thyroid gland of 13-year-old boy with diffuse sclerosing variant of PTC

**Fig. 4:**
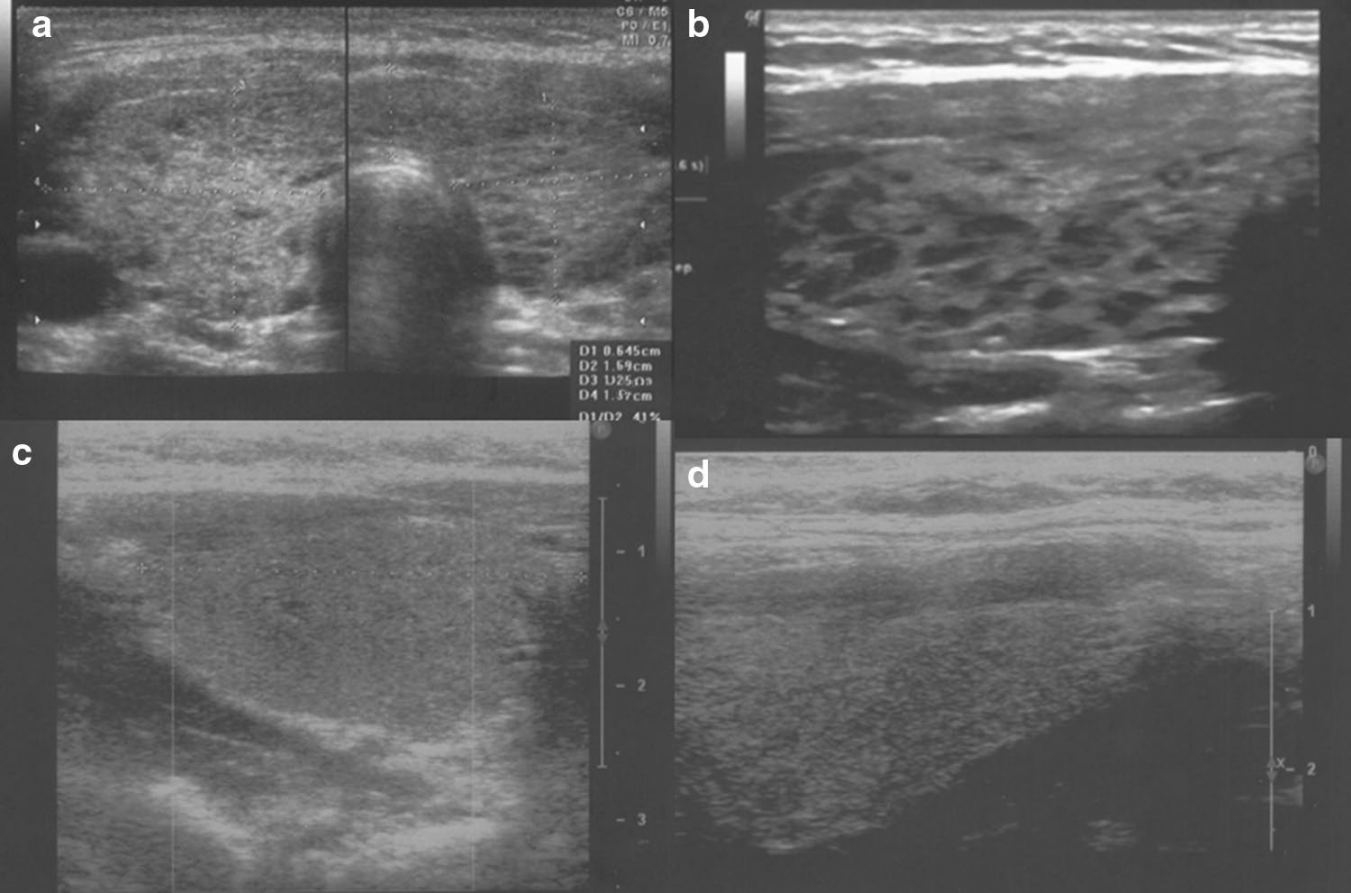
**a**, **b** AIT variant with micronodulations; **c**, **d** AIT variant with diffuse hypoechogenic background

Analysis of the thyroid ultrasound scans from the last 2 years revealed that the following five ultrasound variants of autoimmune thyroiditis were observed in the study group: the most common in 35.2% (63/179)—diffuse thyroiditis with hypoechogenic background and normoechogenic parenchyma (Fig. 1a, b), in 30.2%(54/179)—diffuse thyroiditis with irregular background (Fig. 1c, d), in 18.9% (34/179) nodular variant with normoechogenic irregular background (Figs. 2a–f, 3a–d), in 11.7% (21/179)—micronodulations (Fig. 4a, b), and in 3.9% (7/179)—diffuse hypoechogenic background (Fig. 4c, d; Table 1).

**Table 1:** Thyroid status in different variants of AIT (*p < 0.05 vs diffuse thyroiditis with hypoechogenic background and normoechogenic parenchyma variant with hypothyreosis)

Ultrasound pattern of the thyroid gland	Diffuse thyroiditis with hypoechogenic background and normoechogenic parenchyma			Diffuse thyroiditis with irregular background			Nodular variant and normoechogenic irregular background			Micronodulations			Diffuse hypoechogenic background
*N* (%)	63 (35.2%)			54 (30.2%)			34 (18.9%)			21 (11.7%)			7 (3.9%)
TSH mean [range] uIU/ml (*n*: 0.4–4.0)	62.2 (0.005–692.3)			5.58 (0.001–61.7)			8.5 (0.02–98.91)			16.8 (0.02–248.9)			5.04 (2.04–16)
Thyroid status	T	E	H	T	E	H	T	E	H	T	E	H	E
*N* (%)	8 (12.7%)	11 (17.5%)	44 (69.8%)	15 (27.8%)	19 (35.2%)	20 37%)	4 (11.8%)	18 (52.9%)	12 (35.3%)	1 (4.8%)	11 (52.4%)	9 (42.8%)	7
Compensated *n* (%)	–	–	18 (40.9%)	–	–	11 (55%)	–	–	10 (83.3%)	–	–	7 (77.8%)	–
Overt *n* (%)	8	–	26 (59.1%)	15	–	9 (45%)	4	–	2 (16.7%)	1	–	2 (22.2%)	–
Age years	14.7 (2.7)	14.9 (2.4)	12.8 (2.9)	13.9 (4.1)	14.8 (1.9)	13.0 (3.3)	16.2 (2.9)	15.2 (3.0)	12.7 (2.9)	10.0	14.9 (2.8)	14.1 (1.6)	13.1 3.6)
TSH uIU/ml (*n*: 0.4–4.0)	0.01 (0.0)	3.6 (1.1)	88.1 (153.1)	0.1 (0.2)	2.8 (1.0)	12.7 (12.5)	0.2 (0.4)	2.7 (1.2)	20.0 (26.5)	0.02	2.4 (1.1	36.3 (79.9)	5.0 (5.4)
fT3 pmol/l (n: 3.6–6.8)	27.1 (7.1)	5.4 (0.9)	4.5 (1.6)	17.5 (8.6)	5.3 (1.1)	5.1 (2.1)	15.4 (7.6)	5.6 (0.7)	56 (1.3)	30.8	5.4 (0.5)	4.4 (0.6)	5.2 (0.6)
fT4 pmol/l (*n*: 10–25)	45.3 (31.0)	13.9 (2.4)	9.9 (5.1)	38.5 (21.9)	13.5 (3.7)	12.4 (3.9)	30.6 (13.1)	14.7 (3.9)	11.7 (4.9)	52.0	11.8 (5.7)	10.9 (5.0)	13.2 (1.9)
aTPO IU/ml (*n* < 30)	3572.3 (2757.5)	5467.2 (3615.3)	6041.9 (3526.2)	3040.8 (2792.8)*	1245.5 (1048.4)*	1471.9(1704.0)*	749.5 (1059.2)	1825.7 (2645.7)*	2719.5 (3340.9)*	770	1617.6 (1976.5)*	1922.5 (1670.7)*	487.3 (660.6)
aTG U/ml (*n* < 30)	387.8 (711.8)	829.6 (1148.4)	1880.5 (3082.3)	556.0 (1585.4)*	233.2 (353.9)*	256.9 (415.2)*	49.2 (26.6)	177.7 (216.1)*	384.3 (549.9)*	227.2	634.8 (1484.1)*	1030.5 (2200.2)*	142.9 (133.7)
TRAB U/ml (*n* < 1)	11.1 (7.7)	0.6 (0.5)	1.0 (0.6)	7.9 (7.9)	0.9 (0.2)	0.9 (0.5)	6.8 (2.9)	1.1 (1.1)	1.3 (0.8)	5.8	0.9 (0.1)	4.2 (04.5)	1.2
PTC *n* (%)	–	–	–	–	–	–	1 (25%)	5 (27.8%)	2 (16.6%)	–	–	–	

*T* hyperthyroid, *E* euthyroid, *H* hypothyroid. Data are expressed as mean (SD)

In patients with nodular variant of AIT with normoechogenic irregular background goiter was found in 24/34 patients (70.6%). In this variant of AIT, the mean nodule size was 6.4 mm (2–26 mm). Uninodularity was found in 30 patients (88.2%) and multinodularity in 4 (*n* = 11.8%). FNAB was performed in all patients. In 26/34 patients, FNAB result was assessed as benign (Bethesda II) [16]. PTC was diagnosed after FNAB fulfilling Bethesda V or VI criteria in 8/34 patients (8/34-23.5%, 8/179-4.5%; Table 2) [16]. In patients with PTC, the mean tumor size was 13.3 mm (6–26 mm) (Table 2). In 5 patients with PTC, malignant nodules were hypoechogenic (Figs. 2a, d, 3a, c, d), in 2 patients isoechogenic with hypoechogenic “halo” (Fig. 2c, e, f) and in 1 patient with diffuse sclerosing PTC variant, tiny disseminated hyperechogenic septae and multiple microcalcifications in the whole thyroid gland were seen on US imaging (Fig. 3e, f). In all PTC cases, the echogenicity of thyroid parenchyma was increased when related to the adjacent sternothyroid, sternohyoid, sternocleidomastoid or omohyoid muscles (Figs. 2, 3). In patients with PTC total thyroidectomy with lateral and central lymph nodes, histopathological verification was performed. After the surgery, the clinical outcome in all patients was good and all patients are in remission.

**Table 2:** Characteristics of PTC patients

Patients	Age yrs	Cause of referral	Nodule size (mm)	TNM	PTC variant	131 J	aTPO IU/ml *N* < 30	aTG U/ml *N* < 30	TRAb IU/l *N* < 1	TSH uIU/ml N:0.4–4.0	aTg after surgery
			AACE/ACE/AME Risk group	ATA risk group							
2a,2b	16	Goiter	6	PT1aN0M0	Classic	−	5911.2	300	–	2.12	<20
			Class 2	I							
2c	11	Goiter	12	pT1bN0M0	Follicular with capsule	−	42.94	43.39	–	2.01	<20
			Class 2	I							
2d	18	Goiter	13	pT1bN0M0	Classic	+	<30	<20	5.2	0.02	<20
			Class 3	I							
2e,2f	17	Nodule found on US	10	pT1aN0M0	Classic	−	1271.7	–	–	5.01	<20
			Class 3	I							
3a,3b	17	Nodule found on US	21 × 16 × 26	PT2N1bM0	Follicular	+	853.4	–	0.7	1.02	<20
			Class 3	II							
3c	13	Goiter	8 × 8 × 9	PT1aN1aM0	Classic/follicular	+	126.2	154.3	–	5.05	<20
			Class 3	II							
3d	14	Nodule found on US	7 and 2	PT1aN0M0	7 mm-classic/solid;	−	1300	–	–	4.8	<20
			Class 3	I	2 mm-follicular						
3e,3f	13	Lymph node enlargement	23	PT3N1bM1	Diffuse sclerosing	+	>9000	>8000	1.2	4.12	<20
			Class 3	III							

AACE/ACE/AME US classification system: Class 1-low-risk thyroid lesion; Class 2-intermediate-risk thyroid lesion, Class 3-high-risk thyroid lesion [29]. ATA pediatric risk group: I—low risk, II—intermediate risk, III—high risk [28]

TSH range in different ultrasound classes is presented in Table 1. Analysis of the thyroid status revealed that in our group, 47.5% (85/179) patients were hypothyroid [54.1% (46/85) with compensated hypothyroidism and 45.9% (39/85)—with an overt hypothyroidism], 36.8% (66/179) were euthyroid and 15.6% (28/179) were hyperthyroid at diagnosis (Table 1).

In diffuse thyroiditis with hypoechogenic background and normoechogenic parenchyma variant 69.8% (44/63) patients were hypothyroid at diagnosis (59.1% with an overt and 40.9% with compensated hypothyroidism), 17.5% (11/63) were euthyroid and 12.7% (8/63) hyperthyroid. Significantly higher aTPO and aTG levels were observed in diffuse thyroiditis with hypoechogenic background and normoechogenic parenchyma variant when compared with other AIT variants and aTPO level was also significantly higher than in PTC group.

In diffuse thyroiditis with irregular background, 37% (20/54) were hypothyroid (55% with compensated and 45% with an overt hypothyroidism), 35.2% (19/54) euthyroid and 27.8% (15/54) hyperthyroid.

In nodular variant and normoechogenic irregular background 52.9% (18/34) were euthyroid, 35% (12/34) hypothyroid (83.3% with compensated and 16.7% with an overt hypothyroidism) and 11.8% (4/34) hyperthyroid. Analysis of patients with nodular variant and normoechogenic irregular background revealed that PTC was diagnosed in 27.8% (5/18) of euthyroid, 25% (1/4) of hyperthyroid, and 16.6% (2/12) of hypothyroid patients in this group (all with compensated hypothyroidism). On the other hand, thyroid status analysis of patients with coexistence of AIT and PTC revealed that 62.5% (5/8) PTC patients were euthyroid, 25% (2/8) with compensated hypothyroidism and 1 patient was hyperthyroid. The patient with PTC and thyrotoxicosis was diagnosed at the age of 16.8 years. The cause of referral to the endocrine center was goiter and TSH < 0.02 uIU/ml (*n*: 0.4–4.0), raised fT3-13.4 pmol/l (*n*: 3–8.1) and fT4-31.6 pmol/l (*n*: 10–25). TRab level was 5.2 IU/ml (*N* < 1.0) at diagnosis and 1.0 IU/ml a year later at the time of PTC diagnosis. This patient was treated with decreasing doses of antithyroid drug (thiamasole) until the surgery. The nodule was 13 mm in size, hypoechogenic with microcalcifications, irregular border and increased vascularisation (Fig. 2d; Table 2).

One of the patients with papillary thyroid carcinoma had a diffuse sclerosing variant of PTC with very high unmeasurable aTPO (>9000 IU/ml) and aTG (>8000 IU/ml) levels. After excluding this patient from the PTC group, the mean levels of autoantibodies were as follows: aTPO was 1362.2 IU/ml [range 2081.9–5911.2 IU/ml) and aTG-129.4 IU/ml (range 127.9–300 IU/ml). After the surgery in all PTC patients aTg level was negative.

In micronodulation variant 52.4% (11/21) were euthyroid, 42.8% (9/21) hypothyroid (77.8% with compensated and 22.2% with an overt hypothyroidism) and 4.8% (1/21) hyperthyroid.

Seven patients (3.9%) with positive but relatively low levels of aTPO and aTG levels with diffuse hypoechogenic background on US scans were euthyroid and during further monitoring 3 of them presented a normalization of US echogenicity and aTPO and aTG levels (data not shown). We suspect that this variant corresponds to a transient thyroiditis or to an early phase of AIT.

## Discussion

The analysis of US features seen on the thyroid scans revealed that the following five US variants of autoimmune thyroiditis were observed in our group of 179 paediatric patients: the most common in 35.2% diffuse thyroiditis with hypoechogenic background and normoechogenic parenchyma, in 30.2% diffuse thyroiditis with irregular background, in 18.9% nodular variant with normoechogenic irregular background, in 11.7% micronodulations, and in 3.9% diffuse hypoechogenic background. These ultrasound autoimmune thyroiditis features were already described in previous studies [9, 17–20] but present classification is, to our knowledge, relatively novel in paediatric patients.

In our study group, we have found papillary thyroid carcinoma in 8 patients (8/179 cases) that constituted 4.5% of the whole group. All eight cases of papillary thyroid carcinoma were diagnosed in nodular variant of AIT with normoechogenic irregular background. 23.5% of patients (8/34 cases) with this variant of AIT developed PTC. In 5 patients with PTC malignant nodules were hypoechogenic, in 2 patients isoechogenic with hypoechogenic “halo” and in 1 patient with diffuse sclerosing PTC variant, tiny disseminated hyperechogenic septae and multiple microcalcifications fulfilling whole thyroid gland were seen on US imaging. In all cases, the echogenicity of thyroid parenchyma was increased when related to the adjacent sternothyroid, sternohyoid, sternocleidomastoid or omohyoid muscles. Our results are supported by previous work presenting nodular form of Hashimoto’s thyroiditis within a sonographic background of normal thyroid parenchyma [19]. Usually on US scans in AIT, hyperechoic nodules are more likely to be benign, whereas hypoechoic nodules are more likely to be malignant [19]. In our study, however, two cases of PTC were isoechogenic with hypoechogenic “halo”.

Papillary thyroid carcinoma (PTC) accounts for 90% or more of all childhood DTC cases [8]. According to the data of the Polish National Cancer Registry from 2003 to 2013, new cases of thyroid cancer in patients below 19 years of age constitute 2.3% of all thyroid cancers diagnosed (535 of 22,817 cases in general population) [8] and constitute every second solid neoplasm in girls (following tumors of the central nervous system) and every eighth solid neoplasm in boys [8]. In the last decade, the incidence of thyroid cancer has been increasing at a 6.5% annual rate, making it the fastest growing cancer in the United States [21, 22].

The association between Hashimoto’s thyroiditis and thyroid cancer has been debated and remains an active area of research and controversy [20, 22–24]. If Hashimoto’s thyroiditis could be recognized as a precursor or a risk factor for thyroid cancer, this would have an obvious high clinical impact in paediatric population, given that Hashimoto’s thyroiditis is a rather common disease, with a rising incidence worldwide [22].

Keskin et al. [20] analyzed 300 children with AIT and found the thyroid nodule rate on AIT ultrasound background to be 13%, and the thyroid malignancy rate 0.67%. Corrias et al. [9] reported a thyroid nodule prevalence of approximately 31.5% in 365 pediatric AIT patients; thyroid cancer was present in at least 3% of the patients and in 9.6% of a subset of patients with thyroid nodules. In accordance to these studies, in our work a nodular variant of AIT was found in 18.9% patients, PTC constituted 23.5% of nodular variant cases and in the whole group 4.5%. To summarize as shown above the incidence of PTC in AIT ranges from 0.67 to 4.5% in children. However, as shown in our study, this prevalence of PTC is increased in nodular AIT variant with normoechogenic irregular parenchyma. Based on detailed ultrasound scans analysis, we are convinced that this AIT variant might represent a different type of disease or the parenchymal changes may be secondary to the presence of an occult PTC. The question arises then if in these patients AIT is not secondary to the cancer. Our results are in line with Paparodis findings that the form of AITD pathology (destructive, clinically evident hypothyroid vs. a less destructive, nonclinically evident) might play a role in differentiated thyroid cancer risk [25]. Patients with less destructive AITD (euthyroid, or with compensated hypothyroidism) were described to show a higher risk for differentiated thyroid cancer than were patients with a clear destructive AITD [25]. These observations together with our clinical US findings in paediatric population could provide a support for the hypothesis that AIT might be a secondary event. Whether PTC develops despite autoimmunity (tumor immune-escape mechanism), or due to a target-specific immune response (inflammation, preexisting autoimmunity), or whether AIT develops because of cross-reacting antitumor immunity, needs further research especially in paediatric age range group [26].

From histopathological perspective, it is important to distinguish between diffuse lymphocytic infiltration and focal peritumoral lymphocytic thyroiditis [22]. Hashimoto’s thyroiditis is a diffuse lymphocytic infiltration; therefore, it is considered an independent chronic process and does not signify a reaction to the tumor [22, 26]. The reason for the induced antitumor immune response might be the existence of yet undiagnosed papillary thyroid microcarcinomas [26]. Possibly, these small malignancies that induce a locally defined antitumor immune response, followed by a locally defined inflammation that promotes cross-reactivity, induce an epitope-specific autoimmune response [26]. This hypothesis certainly needs investigation in paediatric population.

The analysis of the thyroid status revealed that in our group 47.5% patients were hypothyroid (54.1% with compensated and 45.9% with an overt hypothyroidism), 36.8% euthyroid and 15.6% hyperthyroid at diagnosis. In accordance with other studies presenting that the majority of AIT children was euthyroid at diagnosis, 62.5% of patients in our group did not receive thyroxine treatment at diagnosis (euthyroid and compensated hypothyroid patients) [15, 17, 27].

The ultrasound variant with diffuse thyroiditis and hypoechogenic background and normoechogenic parenchyma is also defined an atrophic form but the majority of children with this variant presents with goiter [15, 27]. In children, an atrophic form of this variant is not seen; however, we typically observe a reduction of a goiter with the treatment and with time. An atrophic form is usually seen in a transition period and in adulthood. 69.8% of patients with this variant in our group were hypothyroid and the levels of aTPO and aTG were significantly higher than in patients with other variants similarly to other studies [18]. A diffuse fibrosis of the gland in this variant can become evident at a later stage of the disease [15, 19]. Fine echogenic fibrous septae may produce a pseudolobulated appearance of the parenchyma, however, as shown in our study a special notice must be given in differential diagnosis to a diffuse sclerosing variant of PTC presenting tiny disseminated hyperechogenic septae and multiple microcalcifications within the whole thyroid gland [19].

As expected and in accordance to other studies [18], abnormalities in thyroid status in AIT variants with diffuse thyroiditis with hypoechogenic background and normoechogenic parenchyma, with irregular background and with micronodulations were more intense than in nodular variant with normoechogenic parenchyma and in diffuse hypoechogenic background.

On the other hand, patients with nodular variant and normoechogenic irregular background had significantly lower TSH, aTPO and aTG levels than patients with most common AIT variant. The majority of patients with nodular variant as well as with PTC did not receive a thyroxine therapy at diagnosis. This might be in agreement with Paparodis et al. [25] finding of a higher risk for differentiated thyroid cancer in patients with less destructive thyroiditis.

A limitation of the present study is a relatively small number of patients. Multi-center transition studies involving both paediatric and adult patients are needed to evaluate disease and thyroid ultrasound data especially in the light of previous recommendations that thyroid US scans in children with AIT as not changing the treatment, clinical course or outcome, should not be indicated routinely [27] and current recommendations to perform annual thyroid US check-up in all AIT patients [8] or only in patients with a suspicious thyroid examination (suspected nodule or significant gland asymmetry) especially if associated with palpable cervical lymphadenopathy [28].

What our study adds is a proposal to place children with nodular variant and normoechogenic irregular background in the PTC development risk group. Additionally, presented ultrasound classification of AIT variants is relatively novel in paediatric population and represents good basis for further exploration of PTC susceptibility.

## Conclusion

Patients with AIT and nodular variant with normoechogenic irregular background of the thyroid gland on US scans are in the risk group of developing PTC and should be followed up with regular neck US assessment.
